# RETRACTION: Anti‐Inflammatory Activities of *Cinnamomum cassia* Constituent In Vitro and In Vivo

**DOI:** 10.1155/ecam/9841076

**Published:** 2026-03-03

**Authors:** Evidence-Based Complementary and Alternative Medicine

RETRACTION: J.‐C. Liao, J.‐S. Deng, C.‐S. Chiu, W.‐C. Hou, S.‐S. Huang, P.‐H. Shie, and G.‐J. Huang, *“*Anti‐Inflammatory Activities of *Cinnamomum cassia* Constituents In Vitro and In Vivo,” *Evidence-Based Complementary and Alternative Medicine*, vol. 2012 (2012). https://doi.org/10.1155/2012/429320.

The above article, published online on 27 March 2012 in Wiley Online Library (https://wileyonlinelibrary.com), has been retracted by John Wiley & Sons Ltd.

The retraction has been agreed following an investigation of the concerns raised by *Actinopolyspora biskrensis* on PubPeer [1], which identified several concerns related to multiple figures in the paper. More specifically:

**Figure 8a:** Control and Carr panels share identical features with Figure 6a of [2], while the control panel in Figure 4a of [3] appears similar after a horizontal flip. The experimental conditions for the papers differ in the number of animals used and methods of image acquisition.

As a result of the investigation, the data and conclusions of this article are considered unreliable.

The authors have been informed of this decision but did not provide a response.
